# Absence of Deep and Basal Veins Is Common and Clinically Relevant in Sturge-Weber Syndrome

**DOI:** 10.1016/j.pediatrneurol.2025.07.009

**Published:** 2025-07-22

**Authors:** Fatimah M. Albazron, E. Mark Haacke, Ajay Kumar, Sagar Buch, Yang Xuan, Jeong-Won Jeong, Aimee F. Luat, Michael E. Behen, Nore Gjolaj, Csaba Juhász

**Affiliations:** a Department of Psychiatry and Behavioral Neurosciences, Wayne State University School of Medicine, Detroit Michigan; b Translational Imaging Laboratory, Wayne State University School of Medicine, Detroit, Michigan; c Department of Radiology, Wayne State University School of Medicine, Detroit, Michigan; d Department of Neurology, Wayne State University School of Medicine, Detroit, Michigan; e Department of Biomedical Engineering, Wayne State University, Detroit, Michigan; f Wayne State University MR Core Research Facility, Detroit, Michigan; g Division of Neuroradiology, Perelman School of Medicine, University of Pennsylvania, Philadelphia, Pennsylvania; h Department of Pediatrics, Wayne State University School of Medicine, Detroit, Michigan; i Department of Pediatrics, Central Michigan University, Mt Pleasant, Michigan; j Children’s Hospital of Michigan, Detroit, Michigan

**Keywords:** Sturge-Weber syndrome, Magnetic resonance imaging, Susceptibility-weighted imaging, Basal veins, Deep cerebral veins, Motor functions, Stroke-like episodes

## Abstract

**Background:**

Common intracranial vascular abnormalities in Sturge-Weber syndrome (SWS) include leptomeningeal venous malformations (LVMs) and enlarged deep veins. A few small studies have reported absent deep veins in some patients. We used susceptibility-weighted imaging (SWI), a magnetic resonance imaging (MRI) sequence sensitive to detecting small veins, to evaluate deep cerebral veins and the basal vein of Rosenthal (BVR) and assess the radiological correlates and clinical impact of their absence.

**Methods:**

Fifty young subjects, including 30 patients with unilateral SWS and 20 healthy controls, underwent 3T brain MRI prospectively. The presence or absence of the internal cerebral vein (ICV), its two main tributaries, and the BVR were evaluated on SWI in all 50 subjects and correlated with other brain abnormalities and clinical symptoms in the SWS group.

**Results:**

Although deep veins and the BVR were identified bilaterally in all control subjects, absent veins were observed in 70% of patients with SWS: in the SWS-affected hemisphere, absent ICV in 15 (50%), thalamostriate vein in 11 (37%), septal vein in seven (23%), and BVR in nine (30%) patients. Absent contralateral veins were also observed. Absent veins were associated with enlarged and collateral veins. Absent BVR and ICV were associated with extensive LVM, brain atrophy, and worse motor functions (*P* < 0.05); absent BVR was also associated with stroke-like episodes.

**Conclusions:**

Absence of deep and/or basal cerebral veins is common in SWS and is associated with venous vascular anomalies, parenchymal damage, and motor impairment. Absent BVR may also increase the risk for stroke-like episodes.

## Introduction

Sturge-Weber syndrome (SWS) is a rare sporadic congenital neurocutaneous disorder with a prevalence of one in 50,000 births, and most cases are associated with an activating somatic mutation of the *GNAQ* gene in the affected tissues.^1^ SWS is often characterized by a facial capillary malformation known as port-wine stain and intracranial venous vascular abnormalities including leptomeningeal venous malformation (LVM). The latter is considered the hallmark of brain involvement in SWS and is often associated with an enlargement of the deep veins and the choroid plexus.^2–5^ Brain involvement is unilateral in approximately 85% of the cases and often includes atrophy and calcifications.^6^ Brain vascular and parenchymal abnormalities are best visualized with magnetic resonance imaging (MRI), including postcontrast images that can detect the enhancement of the enlarged pial and choroidal blood vessels, whereas susceptibility-weighted imaging (SWI) is superior to visualize the deep vein abnormalities and calcifications.^3,5,7–11^ Common clinical presentations of SWS include early-onset epilepsy, intellectual and motor impairments, learning and behavioral difficulties, and stroke-like episodes, in addition to vascular glaucoma.^2,12–15^

The presumed mechanism of SWS-related brain parenchymal damage involves the impairment of the normal cortical venous outflow leading to venous hypertension and chronic ischemia in the cortex and underlying white matter, with concomitant and progressive brain atrophy and calcification.^6,7,13,16,17^ Extensive LVM and underlying brain atrophy and calcifications have been linked to more severe neurological deficits, including severe epilepsy and motor and cognitive impairments.^7,10,17–19^ However, the early emergence of alternative venous pathways may reduce the detrimental effects of venous flow impairment. Indeed, recent imaging studies utilizing SWI demonstrated that the majority of patients with SWS develop enlarged deep medullary veins (EDMVs) and subependymal veins that can expand postnatally and provide a compensatory venous outflow via the deep venous system.^3,9,20–22^ Extensive multilobar EDMVs were associated with relatively low seizure frequency and better cognitive outcomes in a cross-sectional imaging study, supporting a clinically beneficial compensatory effect of this deep venous remodeling.^9^ In addition, a recent longitudinal study of very young children with SWS also presented some imaging data suggesting that a subarachnoid varicose network can also emerge and expand,^21^ thus providing a collateral venous blood flow from a compromised deep venous system.

Although enlarged deep veins have been commonly reported in SWS, there have also been case reports and small case series showing absent or occluded deep veins in some patients with SWS, mostly involving the internal cerebral vein (ICV) and/or the basal vein of Rosenthal (BVR).^4,21–30^ Most of these studies relied on conventional angiography or clinical MRI techniques (such as postcontrast T1-weighted images), which have limited ability to detect details of the deep veins, especially of those with a small caliber. The prevalence and the potential significance of absent deep veins in SWS remain to be clarified.

In the present study, we utilized MRI with SWI to evaluate deep cerebral and basal veins, along with enlarged veins and parenchymal abnormalities, in a prospectively enrolled cohort of young patients with SWS and healthy control subjects. We had the following goals: to identify normal variations in the deep and basal veins in children and young adults including those reported in the literature^31–39^; to estimate the prevalence of absent and enlarged major deep and basal veins in SWS, along with apparent venous collaterals; to assess the association of such venous abnormalities with other vascular and parenchymal SWS brain abnormalities; and to explore the potential clinical correlates of absent veins.

## Methods

### Participants

Our cohort was selected from a pool of participants enrolled in a prospective clinical imaging research study between 2009 and 2024. The inclusion criteria of the subjects with SWS were (1) age three months to 28 years; (2) unilateral SWS brain involvement established by prior clinical MRI, based on the presence of LVM and/or EDMV; (3) the availability of a prospective 3T MRI brain scan, including an SWI acquisition with good quality; (4) and no prior history of brain surgery. The healthy participants were siblings of the patients with SWS or those with an isolated facial port-wine birthmark but normal brain MRI, with the same age range as the participants with SWS. The control subjects had no prior diagnosis of a neurological disorder, had a normal neurological status upon examination by a licensed pediatric neurologist (A.F. L.), and developed no neurological complications in subsequent follow-up (at least one year). All healthy participants had a good-quality noncontrast brain MRI, including SWI, using the same 3T scanner as the participants with SWS. All study participants who were aged above 30 months underwent a formal age-appropriate neuropsychologic evaluation to establish verbal and nonverbal cognitive, language, and motor functions. Written informed consent was obtained from the parents (for participants aged < 18 years) or the participants (for adults); participants aged 13–18 years signed an assent form. All study procedures were approved by the Institutional Review Board at Wayne State University.

### SWS clinical assessment and cognitive evaluation

Patients were clinically diagnosed with unilateral SWS by a licensed neurologist based on clinical symptoms and clinical brain MRI. A comprehensive evaluation of the previous images and medical reports was conducted by two neurologists (A.F.L. and C. J.). Clinical seizure variables were obtained from medical charts and interviews of parents or adult patients. The assessment of clinical seizure frequency within the year before the study enrollment followed a previously described scoring system,^3,40,41^ which used the following scoring: 0, no history of seizures; 1, history of seizures but seizure free in the last one year; 2, one to 11 seizures per year (i.e., at least yearly but less than monthly seizures); 3, one to four seizures per month; and 4, more than four seizures per month (i.e., at least weekly seizures, on average). Furthermore, all participants underwent a comprehensive battery of age-appropriate neuropsychologic evaluation within 24 hours of their prospective MRI acquisition by a licensed neuropsychologist (N.G. or M.E.B.). In children aged > 30 months, both verbal and nonverbal intelligence quotients were assessed, using the Wechsler Preschool and Primary Scale of Intelligence–Fourth Edition (age 2.5–6 years), Wechsler Intelligence Scale for Children–Fourth Edition (age 6–16 years), or Wechsler Adult Intelligence Scale–Fourth Edition (age >16 years). In addition, fine motor dexterity was assessed using the Grooved Pegboard Test and expressive language functions were evaluated using Clinical Evaluation of Language Fundamentals-4.

### MRI acquisition and processing

A Siemens MAGNETOM Verio 3T scanner (Siemens Medical Solutions, Erlangen, Germany) was used to scan all participants. The MRI acquisition protocol included a T2-weighted turbo spin-echo image (axial, thickness: 4 mm, echo time (TE)/repetition time (TR): 93/6000 milliseconds, acquisition time: 1 minute 14 seconds, flip angle [FA]: 147), fluid-attenuated inversion recovery (axial thickness: 2 mm, TE/TR: 128/9000 milliseconds, acquisition time: 3 minutes 56 seconds, FA: 150), SWI (0.5 × 0.5 × 2.0 mm^3^ voxels, base resolution 448, field of view 224 × 168 × 128 mm, bandwidth 160~410 Hz/pixel, TE 5.1 ms/18 ms, TR: 30 ms, 6/8 partial Fourier along phase encoding, 2 × accelerated parallel imaging (with generalized autocalibrating partially parallel acquisitions) with 24 reference lines, and acquisition time: 5 minutes, FA: 15), and a volumetric T1-weighted magnetization-prepared rapid gradient-echo (MPRAGE) 3D scan (axial, rapid gradient echo, 0.9 × 0.9 × 0.9 mm^3^ voxels, TE/TR: 3/1700 milliseconds, acquisition time: 4 minutes 35 seconds, FA: 9). The processing of the SWI images was performed with the filtered phase multiplication factor of 4 using SPIN software (Signal Processing in NMR; SpinTech MRI, Bingham Farms, MI, USA) to generate minimal intensity projection (MIP) images (8 slices × 2 mm thickness).^42^ Young patients with SWS enrolled before 2021 had moderate sedation, and they also had a postcontrast volumetric MPRAGE sequence with the research MRI acquisition. Other subjects with SWS, who had a nonsedated and noncontrast MRI acquisition performed following the methodology described recently,^43^ also had their recent clinical postcontrast MRIs reviewed.

### Evaluation of the deep and basal veins on SWI

**FIGURE 1.**
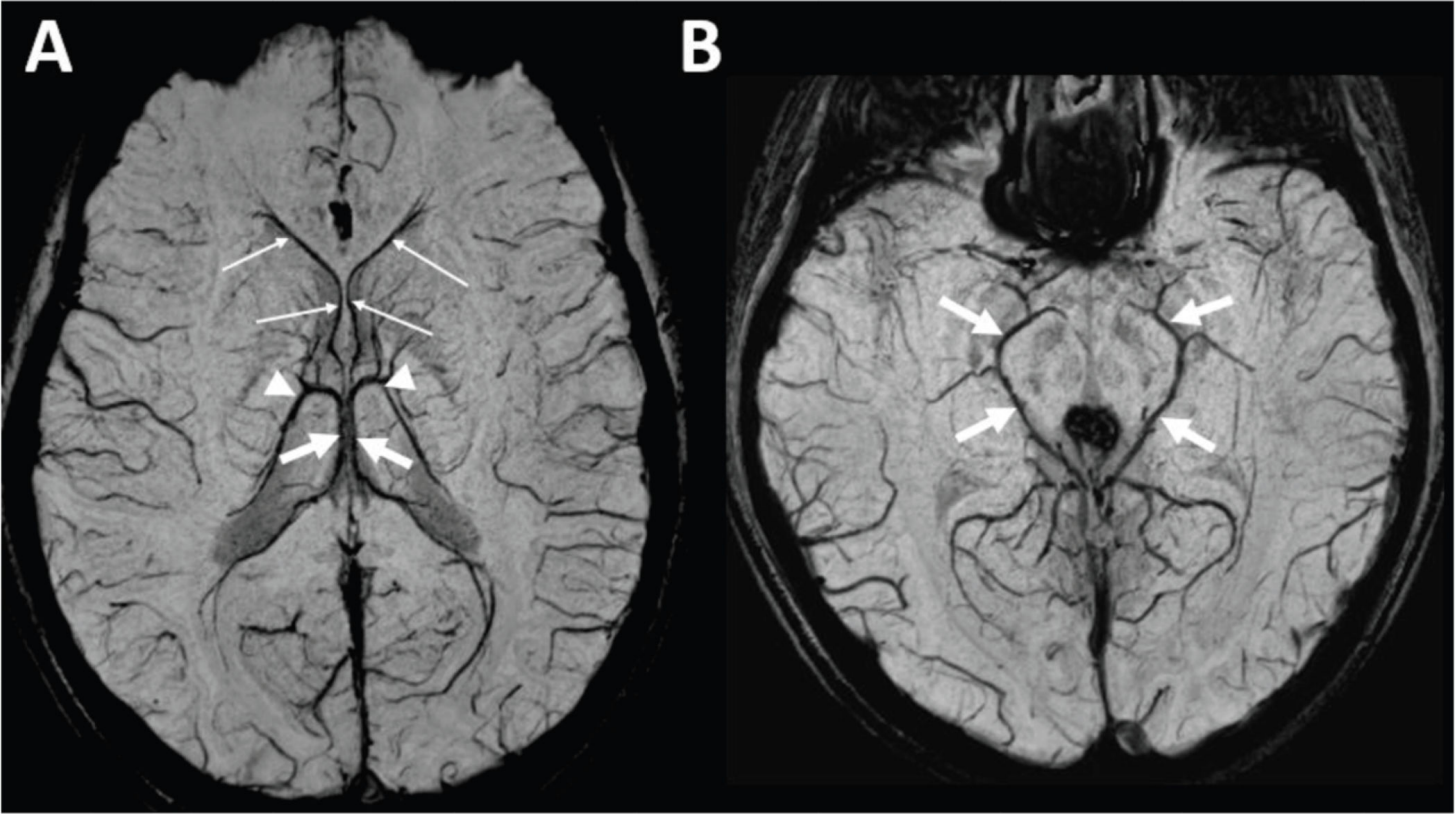
Detection of the major cerebral veins in healthy control subjects on susceptibility-weighted imaging-minimal intensity projection images. (A) The bilateral septal veins are marked by thin arrows at the top, the thalamostriate veins are marked by arrowheads, and the internal cerebral veins are marked by thick arrows. (B) The anterior and the posterior portions of the basal vein of Rosenthal are marked by the top and bottom arrows, respectively.

First, SWI-MIP images of the healthy control subjects were reviewed to evaluate the detectability of three deep cerebral veins: the ICV and its two tributaries, the thalamostriate vein and the septal vein that join to form the ICV, as well as the BVR (Fig 1). The normal variations of these deep veins in both hemispheres were recorded. Subsequently, the presence or absence of these four veins was evaluated in the SWS group. In the cases in which these veins were present, they were categorized as “normal” (similar to the normal variations seen in healthy control subjects) or clearly “enlarged,” whereas in the cases of absent veins, the presence of collateral veins, if any, was also recorded. All images were deidentified and first assessed independently by two investigators (F. M.A. and C.J.) and later jointly to come to an initial consensus. Subsequently, a third investigator (A.K., a board-certified neuroradiologist) re-reviewed the same images independently, and then a final consensus regarding the findings was made after discussing any discrepancies among all three investigators.

### Evaluation of other venous vascular and brain parenchymal abnormalities

The evaluation of additional cerebral venous vascular and parenchymal abnormalities in the SWS group was conducted following a recently reported simple robust scoring system^43^ by two investigators (A.K. and C.J.). Briefly, we recorded the presence (score 1) or absence (score 0) of the following SWS-related brain abnormalities in each of the four lobes in the affected hemisphere: (1) LVM, (2) EDMV, (3) atrophy, and (4) calcification (using the appropriate MRI sequences: postcontrast T1/MPRAGE for LVM, SWI-MIP for EDMV, T2 for atrophy, and SWI high-pass filtered phase images for calcification). In addition, the extent of EDMVs (where present) was further assessed using a more detailed scoring system.^3^ In brief, the number of EDMVs was assessed on SWI-MIP images in each one of the five EDMV territories (frontal, central, parietal, temporal, and occipital) and scored on a scale of 0–3. The scores were then summed across the five regions, providing a score range from 0 to 15; these summed hemispheric EDMV scores had a strong test-retest reliability in our previous study (intraclass correlation coefficient: 0.96; 95% confidence interval: 0.89–0.99).^3^

### Statistical analysis

Normality of the clinical and MRI variables was tested using the Shapiro-Wilk test. The prevalence of absent and enlarged deep and basal veins was characterized using descriptive statistics. Since the majority of the variables showed non-normal distribution, group values are expressed as median and quartiles (25%−75%), and group comparisons were done by Mann-Whitney *U* test. The chi-square test was used to analyze the relationship between binary categorical variables. Multivariate analysis was performed by binary logistic regression. *P* values < 0.05 were considered to be significant after Bonferroni correction, where applicable. All statistical analyses were conducted using SPSS 29.0 (IBM Corp., Armonk, NY, USA).

## Results

### Participant demographics and clinical variables

A total of 50 subjects met our eligibility criteria: 30 patients with SWS (19 females; age range: 1–24 years, median: 13.0 [4.4–19.8] years) and 20 healthy control subjects (eight females; age range: three months to 28 years, median: 13.0 [7.9–20.0] years). Clinical characteristics, including cognitive and motor scores, in the right and left SWS subgroups, are shown in Table 1. Seventeen patients (57%) had right-sided SWS, based on the side of LVM and/or EDMV (two subjects had no detectable leptomeningeal enhancement on postcontrast images or SWI but had EDMVs). Twenty-one patients with SWS (70%) had a facial port-wine birthmark, whereas nine patients had only brain involvement. Seizure history was reported in 28 patients (93%), with a median age of seizure onset of 0.6 (0.3–1.9) years, a mean duration of epilepsy of 11.0 (2.4–19) years, and a median seizure frequency score of 1 (1–2). Nine patients (30%) had a history of stroke-like episodes. As expected, all healthy control subjects were measured within normal limits on all intellectual, language, and motor indices (data not shown).

### Evaluation of deep and basal cerebral veins in the control group

**FIGURE 2.**
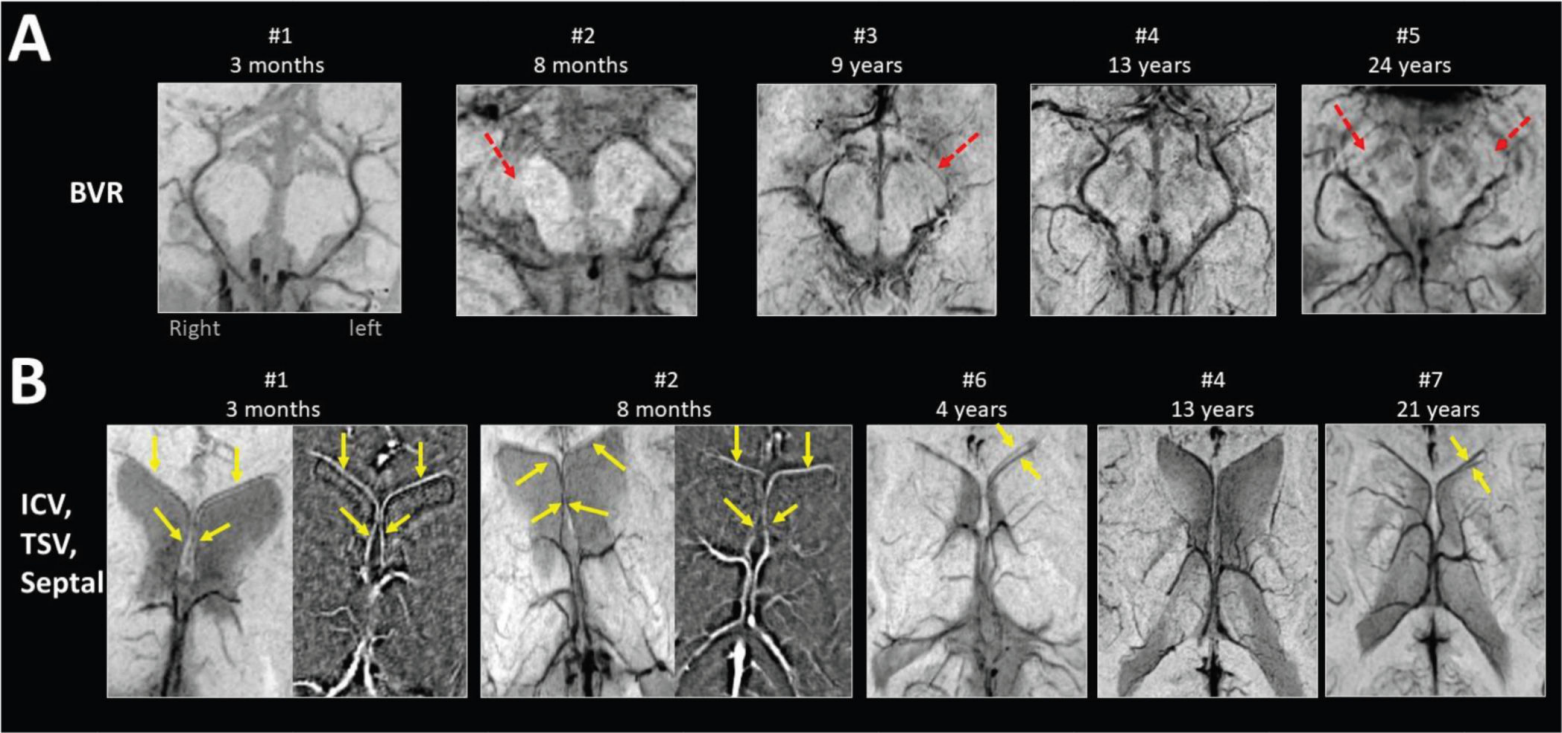
Axial susceptibility-weighted imaging-minimal intensity projection (SWI-MIP) images of anatomic variations in healthy subjects of different ages. (A) Five healthy control subjects with different BVR variants. Subjects 1 and 4: both the anterior and the posterior segments of BVR are present, symmetric; subject 2: absence of the right anterior segment of BVR (arrow); subject 3: absence of the left anterior segment of BVR (arrow); subject 5: bilateral absence of the anterior segments of BVR (arrows). (B) Variants of thalamostriate and septal veins; subjects 1, 2, and 4: symmetric branching of thalamostriate vein and a single branch of the septal veins (yellow arrows highlight the septal veins on SWI-MIP and filtered phase images in the two youngest subjects, where the additional phase images helped to confirm the presence of both septal veins); subject 6: symmetric branching of thalamostriate vein and a double branching of the left septal vein (double arrows); subject 7: asymmetric branching of the thalamostriate veins and double branching of the left septal vein (double arrows). BVR, basal vein of Rosenthal; ICV, internal cerebral vein; TSV, thalamostriate vein.

All four cerebral veins were clearly visible and identifiable bilaterally on SWI in all healthy control subjects, without any asymmetric enlargement of any of these veins (see examples at different ages in Fig 2). Variations of these veins included absence of the anterior portion of the BVR (40%; n = 8), a branching of the septal vein in one hemisphere (35%; n = 7), and an asymmetric branching of the thalamostriate vein (15%; n = 3).

### Abnormal leptomeningeal and medullary veins and parenchymal MRI abnormalities in the SWS group

SWS brain abnormalities included LVM (28 of 30, 93%), EDMV (27 of 30, 90%), atrophy (27 of 30, 90%), and calcification (20 of 30, 67%) in at least one lobe of the affected hemisphere. The prevalence of these abnormalities was not different in those with right versus left hemispheric SWS (*P* > 0.3 in all group comparisons).

### Absent cerebral veins in the SWS group

Only nine of the 30 patients with SWS (30%) had all four cerebral veins clearly visible and identifiable in both hemispheres; the remaining 21 patients (70%) showed various combinations of absent veins (Table 2).

**FIGURE 3.**
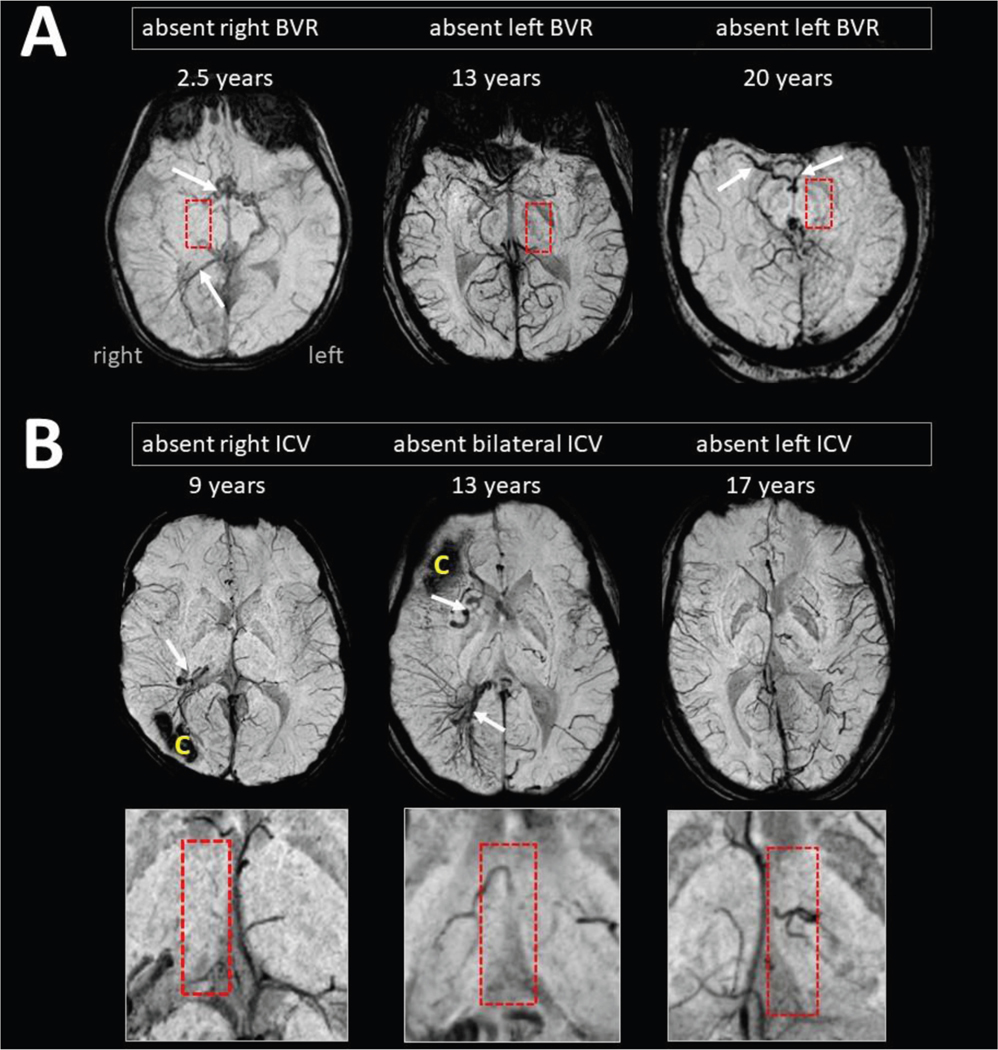
Axial susceptibility-weighted imaging-minimal intensity projection images for venous abnormalities in Sturge-Weber syndrome (SWS). The years refer to the ages of the patients. (A) Three examples of basal vein of Rosenthal (BVR) anomalies; the areas of absent BVR are indicated within the box, and collateral veins are pointed out by arrows. (B) Three examples of anomalies affecting the internal cerebral vein (ICV). The areas of absent ICV are indicated in the box on the enlarged panels (bottom row). Absent thalamostriate and septal veins are also observed. Collateral veins are pointed out by arrows. Dark areas with a letter C represent subcortical calcifications.

The absence of the ipsilateral ICV was more than twice as prevalent in the right-sided SWS as in the left-sided SWS (11 of 17 [65%] vs four of 13 [31%], *P* = 0.015), whereas the absence of the other veins showed no side differences. Absent veins were also observed in the contralateral hemisphere (not showing other signs of SWS brain abnormality) in several cases, often along with the absence of ipsilateral veins (Fig 3).

### Enlarged deep veins and collaterals

**FIGURE 4.**
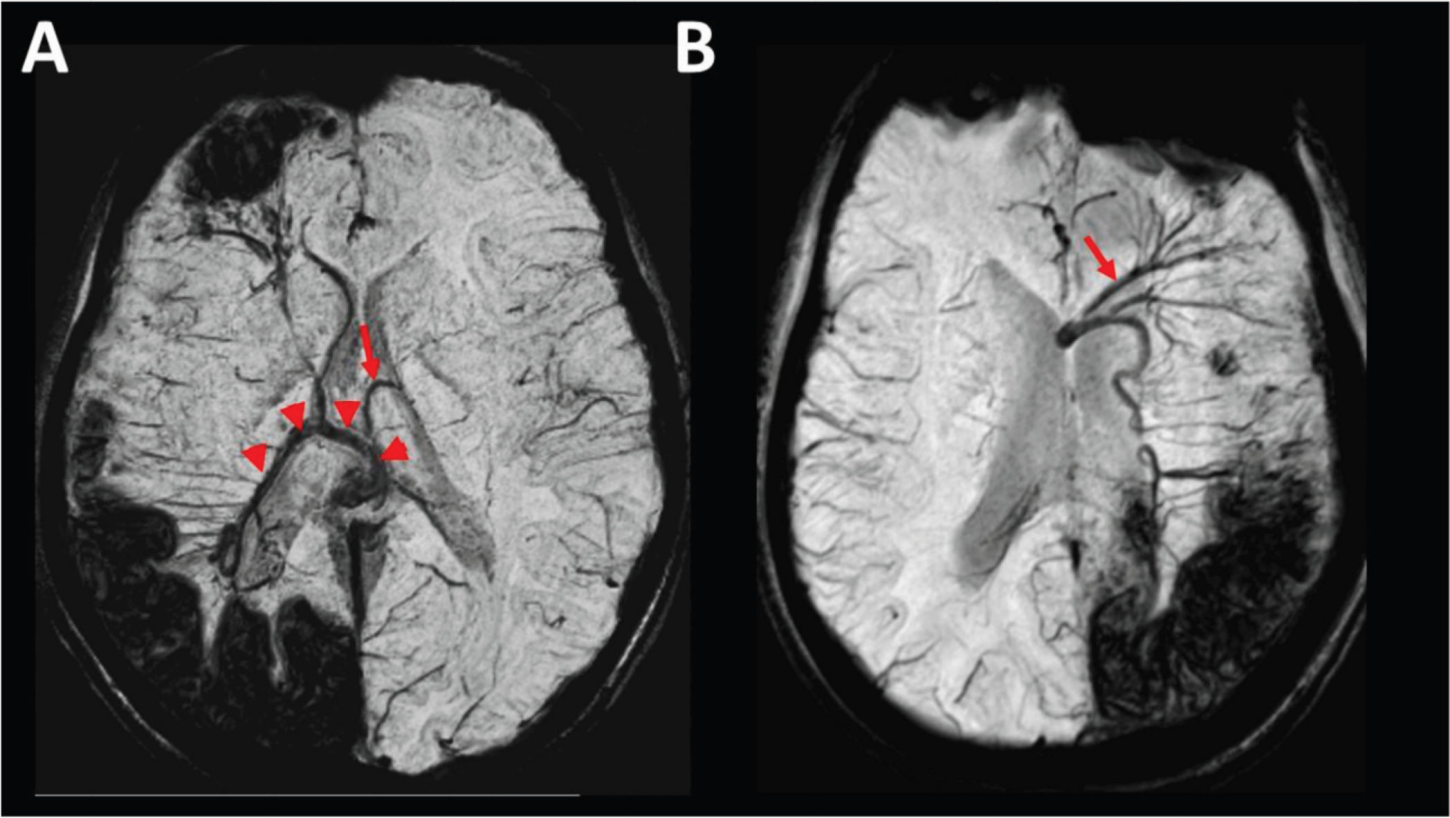
Examples of massively enlarged deep veins. (A) Axial susceptibility-weighted imaging-minimal intensity projection (SWI-MIP) image from a 13.5-year-old patient with extensive right-hemispheric Sturge-Weber syndrome brain involvement including multilobar leptomeningeal venous malformation, atrophy, and several enlarged medullary veins. Normal internal cerebral veins (ICVs) were not visualized on either side; instead, a massively enlarged vein (likely the left ICV) was detected (arrowheads), which was connected to enlarged medullary and subependymal veins from the right frontal and posterior regions, crossing the midline, where it was also connected to the left thalamostriate vein (arrow) and converging posteriorly on the vein of Galen. (B) Axial SWI-MIP image from a 21-year-old patient with a massively enlarged left septal vein (arrow) in a case of a bilateral absence of the ICVs, as well as an absence of the contralateral septal vein.

Additionally, in 18 of the 21 patients (86%), where some cerebral veins were absent, collateral veins were also detected (Fig 3). In cases of absent ICVs, the collaterals often appeared to involve atypical/enlarged intrathalamic veins with variations and connections that were not observed in the healthy control subjects and were not consistent with the normal variations reported for the superior thalamic vein^44,45^ (see Supplementary Material). The absence of the ipsilateral ICV was associated with a large contralateral ICV connected to EDMVs in the affected hemisphere in two patients (see example in Fig 4A). Asymmetric enlarged veins were most common in the septal veins (37%; 11 of 30), including 10 patients with absent septal vein on the contralateral side (see example in Fig 4B).

### Association of absent ICV and BVR with other radiological features and clinical variables

The radiological and clinical variables in patients with SWS with absent versus present BVR and ICV are shown in Tables 3 and 4, respectively. The BVR was absent in only one subject contralateral to the affected hemisphere; therefore group comparisons were made only for the absent ipsilateral BVR. The absence of the ipsilateral BVR was associated with greater extent of the LVM (*P* = 0.009), greater EDMV scores (*P* = 0.007), and more extensive atrophy (*P* = 0.004), as well as lower motor function scores (*P* = 0.005). The absence of the contralateral ICV was also associated with greater extent of the LVM (*P* = 0.008) and lower motor scores (*P* < 0.001). In addition, the prevalence of stroke-like episodes was almost threefold greater in those with absent ipsilateral BVR (five of nine, 55%) when compared with those with present ipsilateral BVR (four of 21, 19%; *P* = 0.046).

To evaluate if the association of absent BVR and motor impairment is independent of other predictors of motor dysfunction, first nonbinary MRI and clinical predictors were compared between patients with SWS showing motor T-scores ≤0 (i.e., severe motor impairment, n = 12) versus those with scores >0 (n = 16). In the second step, the two most significant nonbinary predictors of severe motor impairment (the extent of atrophy [*P* = 0.001] and LVM [*P* = 0.004]) were entered in a binary logistic regression analysis together with the presence/absence of ipsilateral BVR. In this analysis, the absence of BVR was the only significant predictor of severe motor impairment (*P* = 0.046), whereas the other two MRI predictors were not significant (atrophy: *P* = 0.44, LVM: *P* = 0.53). For stroke-like episodes, none of the other (nonbinary) imaging or clinical variables showed a significant difference in univariate analysis; therefore no further multivariate testing was done. The absence of contralateral ICV was not associated with motor scores in a similar multivariate analysis.

## Discussion

In this study, we investigated the prevalence and the potential clinical impact of absent deep and basal veins in 30 young patients with unilateral SWS using SWI at 3T. This technique was able to detect such veins reliably in 20 healthy control subjects, regardless of their age, whereas the SWS group showed a high prevalence (70%) of absence of one or multiple deep and/or basal veins. Surprisingly, these absences were not always confined to the hemisphere with other vascular (LVM, EDMV) or parenchymal SWS abnormalities but were also present in the contralateral hemisphere in some patients. Absent veins were associated with more severe brain vascular and parenchymal abnormalities, and most patients with an absent vein also had enlarged and/or collateral veins. This finding indicates the presence of complex venous abnormalities that likely emerged during early brain development, although the timing of these remains to be determined. Absent BVR was associated with poor motor function and stroke-like episodes, independent of other brain abnormalities. Overall, these findings indicate that absent deep and/or basal veins are a more common vascular pathology in SWS than previously appreciated and that they can have direct clinical relevance, especially for motor outcomes.

Deep and basal veins in the healthy human brain have a high anatomic variability,^35,36^ with several previous studies reporting the common anatomic variations of the ICV,^36,38,46^ thalamostriate vein,^32^ and septal vein,^37^ as well as the physiologic variants of the BVR.^31^ We were able to detect previously reported variants in our control subjects as young as three months. This fact provides the foundation for detecting the major deep and basal cerebral veins to correctly interpret the abnormal variants. Thus, it is unlikely that the observed venous abnormalities, including absent veins, were due to technical reasons or physiologic variations in the SWS group.

The main novelty of our study lies in the detection of high prevalence of the absence of at least one deep and/or basal vein (70%) in young patients with SWS. Although absent or occluded deep veins have been reported occasionally in previous case reports and small SWS case series,^23–30^ a high prevalence has not been appreciated in the literature. However, previous studies did not utilize SWI to assess these veins systematically. Interestingly, the absence of the ipsilateral ICV was more than twice as common in right-sided than in left-sided SWS (65% vs 31%), and this right-hemispheric prevalence is similar to our previous finding of the higher prevalence of extensive EDMVs in the right-sided SWS.^3^ The cause of this right-sided predominance remains unknown and needs to be confirmed in larger cohorts. Nevertheless, absent BVR and ICV was associated with more extensive LVM and EDMVs, indicating that the venous vascular abnormalities may be related to and reflect different aspects of the same pathophysiology and cerebral venous remodeling in SWS. Although EDMVs and other enlarged cerebral veins may provide effective compensatory venous outflow, the absence of BVR and, to a lesser degree, the absence of ICV were associated with more extensive multilobar atrophy and calcification, indicating a potential impact of these venous abnormalities on parenchymal damage.

An unexpected aspect of our findings was the occasional absence of deep veins in the contralateral “unaffected” hemisphere (which showed no LVM/EDMV and/or parenchymal abnormalities), especially for the ICV, which was absent bilaterally in five patients. The ICV is a major deep cerebral vein that typically drains the venous blood from the thalami and periventricular white matter. The bilateral absence of ICV without severe bilateral parenchymal (e.g., thalamic) damage, along with the common presence of apparent collateral veins, strongly suggests the developmental nature of absent ICVs. Although progressive venous occlusion has been documented in SWS,^27^ progressive bilateral thrombosis of the ICV during the postnatal disease course would likely result in bilateral venous infarcts and associated severe symptoms. Absent deep veins along with common collaterals (including apparent collateral thalamic veins associated with absent ICV), observed in this study, indicate a complex venous abnormality including development of venous compensatory channels that may limit the damage of the affected brain regions. Nevertheless, early postnatal expansion of deep venous collaterals is possible, as documented in our previous reports in young children with SWS.^9,20^ It should be noted that although thalamic infarcts are not (or only exceptionally) observed on clinical MRI of patients with SWS, we have previously documented thalamic diffusion and metabolic abnormalities in the affected hemisphere, and the severity of these was associated with cognitive function in the left-hemispheric subgroup.^47^ Future studies could evaluate if the absence of ICV or other deep veins is related to such thalamic microstructural or functional abnormalities.

Our data also revealed a direct association between absent BVR and lower motor function scores as well as a higher prevalence of stroke-like episodes manifesting with temporary motor weakness. The BVR is a main vein along the medial temporal lobe, with a wide venous draining territory that includes the insula, mesial temporal lobe structures, the hypothalamus, parts of the striatum and thalamus, and the midbrain. Impaired venous outflow from some of these structures may affect motor functions, but the mechanism of motor dysfunction in SWS is likely more complex and may involve both cortical and subcortical abnormalities. The use of aspirin was suggested to be effective to prevent some of the stroke-like episodes,^48^ and it would be interesting to evaluate if the effectiveness of aspirin is related to the patency of BVR or deep veins in young patients with SWS. Details and timing of aspirin use were not consistently documented in several of our patients thus precluding such an analysis in the current cohort; future studies could address this issue.

Absent ICV contralateral to the hemispheric SWS involvement was associated with lower age at seizure onset as well as lower expressive language and nonverbal intelligence quotient scores, although the differences were not significant after correcting for multiple comparisons. This lack of statistical significance may be due to the limited statistical power of the small subgroups; also, the effect of absent veins on cognitive/language scores may be moderated by functional reorganization and plasticity of the developing brain, including transfer of language functions from the left to the right hemisphere.

Our study has several limitations. As common in single-center studies of a rare disorder, our sample size was limited, and this could limit the statistical power, especially in subgroup comparisons. Also, the brain MRIs were done at a wide age range at different stages of the disease course. Nevertheless, despite the small control group, it was reassuring to document the high sensitivity of SWI to detect normal veins and their physiologic variations, even in young subjects. This fact suggests the feasibility of this technique to detect deep and basal venous abnormalities in infants with suspected SWS. Such studies in the future would be useful to determine how much of the observed complex venous abnormalities are present shortly after birth and which of those emerge or expand during the early disease course.

In conclusion, we report here a complex venous abnormality in the majority of patients with SWS that involves absent, enlarged, and collateral veins. The ultimate parenchymal damage caused by these venous abnormalities, and their clinical impact, is likely determined by the combined effects of these abnormalities, including their timing and extent. Absent BVR and/or ICV may serve as an early imaging marker indicating an enhanced risk for neurological complications. However, more studies are needed to further investigate the integrity of cerebral veins and the emergence of these complex venous abnormalities in young patients with SWS, including presymptomatic infants, where preventive measures may have the strongest impact to alter the development of symptoms and optimize clinical outcome.

## Supplementary Material

MMC1

Supplementary data

Supplementary data related to this article can be found at https://doi.org/10.1016/j.pediatrneurol.2025.07.009.

## Figures and Tables

**TABLE 1.** Clinical Characteristics of Patients With Right and Left SWS Brain Involvement

Clinical Variables	Number of Values (Right/Left)	Right SWS	Left SWS	*P* Value

Gender (female/male)	17/13	12/5	7/6	0.45
Age (years)	17/13	13.1 (8.6–19.8)	13.0 (2.6–18.5)	0.65
Age at seizure onset (years)	15/13	0.8 (0.5–1.5)	0.5 (0.3–4.2)	0.72
Epilepsy duration (years)	15/13	12.2 (4.8–19.2)	4.6 (2.2–17.3)	0.23
Seizure frequency score	17/13	1 (1–2)	2 (1–2)	0.07
Stroke-like episodes (No.)	17/13	6/17 (35%)	3/13 (23%)	0.47
VIQ	16/10	89 (82–106)	92 (79–100)	0.78
NVIQ	16/9	90 (75–99)	82 (77–95)	0.80
Expressive language score	14/11	46 (39–51)	42 (31–45)	0.16
Motor score (ipsilateral)	15/9	43 (21–49)	45 (23–53)	0.52
Motor score (contralateral)	15/9	4 (−42 to 28)	20 (−50 to 55)	0.30

Abbreviations:

IQ = Intelligence quotient

NVIQ = Nonverbal IQ

SWS = Sturge-Weber syndrome

VIQ = Verbal IQ

Ipsilateral/contralateral: hand motor scores ipsilateral/contralateral to the SWS brain involvement. Motor functions are expressed as T-scores; in patients in whom the test could not be performed due to severe motor weakness of the hand, the scores were set to − 50 (i.e., lower than any score in testable patients).

For nonbinary variables, median and quartiles (25%−75%) are indicated. For some clinical variables, the number of values was <30, as some neurocognitive scores were not available in all patients (e.g., due to young age), and two patients had no history of clinical seizures.

**TABLE 2.** The Number of Absent and Present Cerebral Veins in Left- and Right-Hemispheric SWS

Veins Assessed	Right SWS (N =17)				Left SWS (N =13)			
	Ipsilateral		Contralateral		Ipsilateral		Contralateral	
	Absent	Present (Enlarged)	Absent	Present (Enlarged)	Absent	Present (Enlarged)	Absent	Present (Enlarged)

ICV	11	6 (4)	4	13 (1)	4	9 (2)	3	10 (0)
Thalamostriate vein	8	9 (2)	4	13 (1)	3	10 (2)	3	10 (0)
Septal vein	5	12 (7)	6	11 (0)	2	11 (4)	4	9 (0)
BVR	6	11 (2)	1	16 (1)	3	10 (1)	0	13 (0)

Abbreviations:

BVR = Basal vein of Rosenthal

ICV = Internal cerebral vein

SWS = Sturge-Weber syndrome

Absence of the major cerebral veins was mostly ipsilateral to the side of the brain involvement, with the ipsilateral ICV most commonly affected (n = 15, 50%), followed by the thalamostriate vein (n = 11, 37%), BVR (n = 9, 30%), and the septal vein (n = 7, 23%) (Table 2); see examples in Fig 3.

In case of present veins, the number of patients with enlarged veins is indicated in parentheses.

**TABLE 3.** Comparison of Radiological and Clinical Variables (Median and 25%−75% Quartile Range) in Those With Absent Versus Present BVR Ipsilateral to the SWS Brain Involvement

MRI and Clinical Variables	Absent BVR (N = 9)	Present BVR (N = 21)	*P* Value

SWS-related MRI abnormalities			
LVM extent (no. of lobes)	4 [3–4]	2 [1–3.5]	0.009*
Enlarged deep medullary vein score	8 [4.5–13]	4 [2–5]	0.007*
Atrophy extent (no. of lobes)	4 [2.5–4]	1 [1–2.5]	0.004*
Calcification extent (no. of lobes)	1 [0–4]	1 [0–2]	0.42
Clinical variables			
Age at seizure onset (year; 9/19)	0.6 [0.3–2.0]	0.6 [0.3–2.0]	0.63
Epilepsy duration (year, 9/19)	11.5 [4.5–20.0]	8.7 [2.3–18.4]	0.41
Seizure frequency score	1 [1–1.5]	2 [1–2]	0.60
Motor score (contra) (9/19)	−50 [−50 to −4]	19 [4–51]	0.005*
Verbal IQ (8/18)	90 [73–105]	90 [83–100]	0.69
Nonverbal IQ (8/17)	94 [73–99]	82 [78–94]	0.32
Expressive language score (8/17)	43 [32–55]	43 [39–49]	0.93

Abbreviations:

BVR = Basal vein of Rosenthal

IQ = Intelligence quotient

LVM = Leptomeningeal venous malformation

MRI = Magnetic resonance imaging

SWS = Sturge-Weber syndrome

For the clinical variables, the number of available values for each subgroup is indicated in parentheses (where not all subjects had the variable available).

* Significant *P* values after Bonferroni correction.

**TABLE 4.** Comparison of Radiological and Clinical Variables (Median and 25%−75% Quartile Range) in Those With Absent Versus Present ICV Ipsilateral and Contralateral to the SWS Brain Involvement

MRI and Clinical Variables	Ipsilateral ICV			Contralateral ICV		
	Absent (N = 15)	Present (N = 15)	*P* Value	Absent (N = 7)	Present (N = 23)	*P* Value

SWS-Related MRI Abnormalities						
LVM extent (number of lobes)	3 [2–4]	2 [1–4]	0.60	4 [4–4]	2 [1–3]	0.008*
Enlarged deep medullary vein score	5 [4–11]	3 [2–5]	0.056	8 [4–12]	4 [2–6]	0.069
Atrophy extent (number of lobes)	2 [1–4]	2 [1–4]	0.68	4 [3–4]	2 [1–3]	0.014
Calcification extent (number of lobes)	1 [1–3]	0 [0–2]	0.067	2 [1–3]	1 [0–1]	0.019
Clinical variables						
Age at seizure onset (year; 14/14 and 7/21)	0.7 [0.4–2.1]	0.6 [0.3–2.0]	0.67	0.3 [0.1–1.3]	0.8 [0.5–2.7]	0.08
Epilepsy duration (year) (14/14 and 7/21)	11.3 [2.4–19.4]	9.8 [2.3–18.7]	0.73	12.0 [2.4–20]	8.7 [2.4–16.5]	0.47
Seizure frequency score (15/15 and 7/23)	1 [1–2]	2 [1–2]	0.07	2 [1–2]	1 [1–2]	0.77
Motor score (contra) (15/13 and 7/21)	4 [–50–51]	8 [–29–29]	0.86	−50 [−50 to −30]	19 [2–51]	<0.001*
Verbal IQ (13/13 and 7/19)	86 [77–103]	92 [87–100]	0.45	84 [72–108]	91 [85–100]	0.46
Nonverbal IQ (13/12 and 7/18)	88 [74–98]	85 [77–96]	0.93	80 [70–92]	91 [81–100]	0.09
Expressive language score (14/11 and 5/20)	42 [35–49]	46 [42–49]	0.37	32 [29–41]	45 [42–50]	0.015

Abbreviations:

ICV = Internal cerebral vein

IQ = Intelligence quotient

LVM = Leptomeningeal venous malformation

MRI = Magnetic resonance imaging

SWS = Sturge-Weber syndrome

For the clinical variables, the number of available values for each subgroup is indicated in parentheses.

* Significant *P* values after Bonferroni correction.
