# Supplementary material for: Absence of Deep and Basal Veins Is Common and Clinically Relevant in Sturge-Weber Syndrome
Source: Pediatr Neurol. Author manuscript; Available in PMC 2026 Jan 3. (PMC12762158; doi:10.1016/j.pediatrneurol.2025.07.009)

**Supplementary material**

Visualization of intrathalamic veins on SWI-MIP images in the healthy controls (**Suppl. Figure 1**) and in SWS patients with absent internal cerebral vein (ICV) (**Suppl. Figure 2**).

**Suppl. Figure 1**. SWI-MIP images of the thalamus from 12 healthy control subjects. The location of the superior thalamic veins (STVs), the main draining vein of the thalamus, are indicated with red arrows. The course of these STVs showed physiologic variations, consistent with variants reported previously [43]. The most common variants are consistent with drainage into the posterior or anterior portion of the ICV, which has been reported in 2/3 of healthy controls [44].


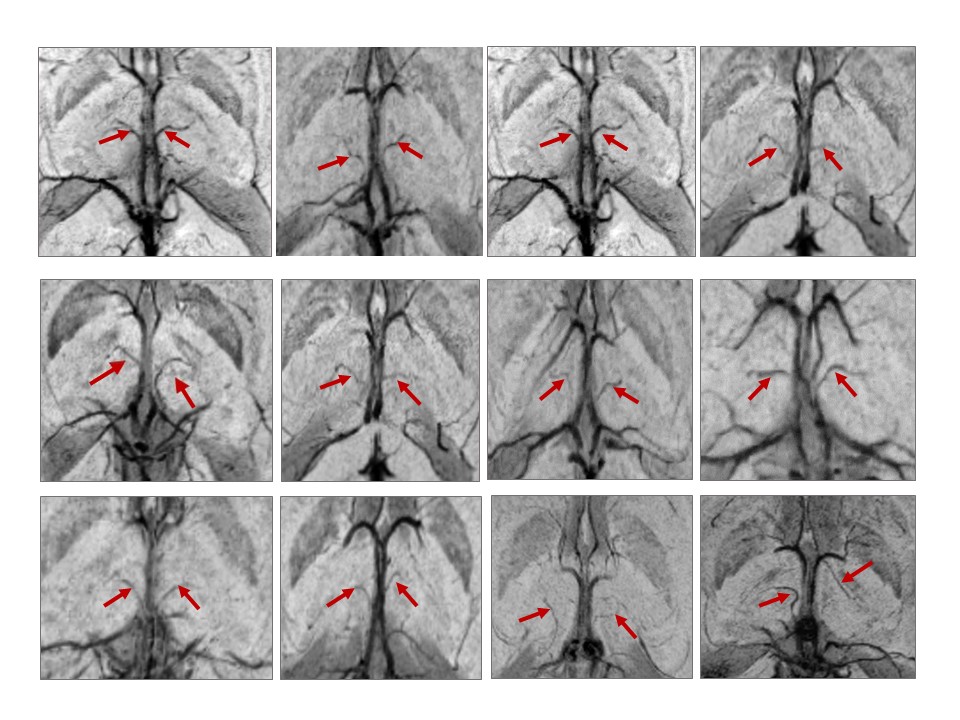


**Suppl. Figure 2**. SWI-MIP images of the thalamus from 9 patients with SWS and absent ICV, including those with absent bilateral (Bi) or unilateral (right or left) ICV. The yellow arrows point out intrathalamic veins on the side of absent ICVs, showing their variable, alternative drainage routes in the absence of the ICV.


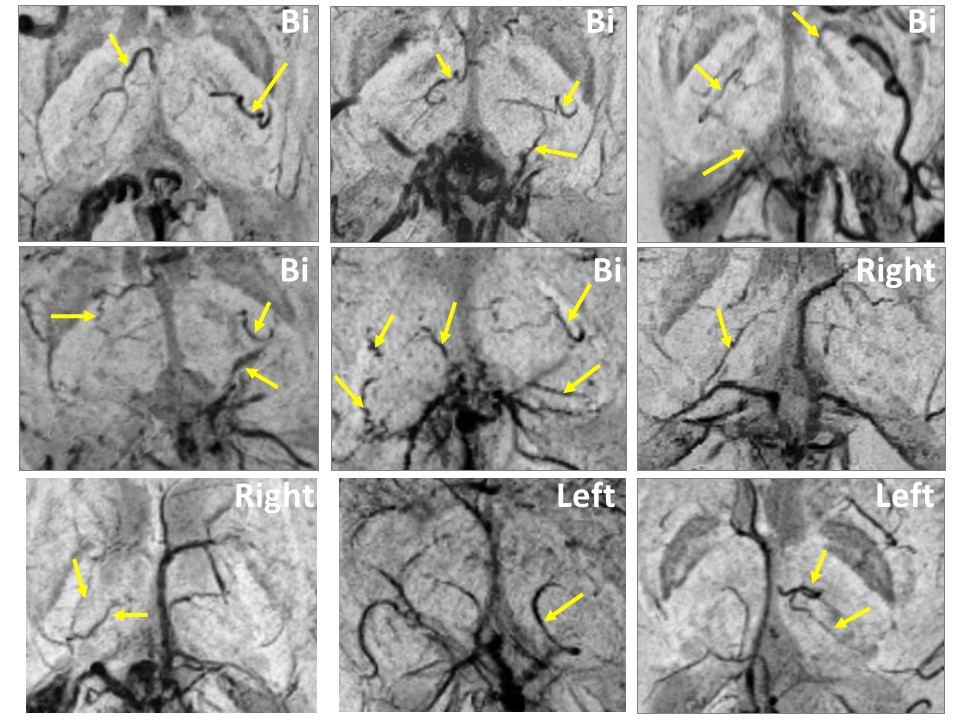

Supplement: MMC1 [file NIHMS2099451-supplement-MMC1.docx]
